# Patterns of recurrence in oesophageal cancer following oesophagectomy in the era of neoadjuvant chemotherapy

**DOI:** 10.1002/bjs5.30

**Published:** 2018-03-15

**Authors:** W. R. C. Knight, J. Zylstra, M. Van Hemelrijck, N. Griffin, A. E. T. Jacques, N. Maisey, C. R. Baker, J. A. Gossage, J. Largergren, A. R. Davies

**Affiliations:** ^1^ Department of Surgery Guy's and St Thomas' Oesophago‐Gastric Centre London UK; ^2^ Division of Cancer Studies Division of Cancer Studies King's College London London UK; ^3^ Translational Oncology and Urology Research (TOUR) Division of Cancer Studies King's College London London UK; ^4^ Department of Radiology Guy's and St Thomas' Hospital London UK; ^5^ Department of Oncology Guy's and St Thomas' Hospital London UK; ^6^ Gastrointestinal Research Unit, Department of Molecular Medicine and Surgery Karolinska Institute, Stockholm, Sweden, on behalf of the Guy's and St Thomas' Oesophago‐Gastric Research Group

## Abstract

**Background:**

Tumour recurrence following oesophagectomy for oesophageal cancer is common despite neoadjuvant treatment. Understanding patterns of recurrence and risk factors associated with locoregional and systemic recurrence might influence future treatment strategies.

**Methods:**

This was a cohort study involving patients undergoing resection for adenocarcinoma or squamous cell carcinoma of the oesophagus between 2000 and 2014. Clinicopathological factors associated with locoregional and systemic recurrence were analysed using multivariable logistic regression to determine odds ratios (ORs) and 95 per cent confidence intervals.

**Results:**

Some 698 patients were identified. Lymphovascular invasion (OR 2·09, 95 per cent c.i. 1·18 to 3·71) and preoperative stenting (OR 3·70, 1·34 to 10·23) were independent risk factors for isolated locoregional recurrence. Pathological nodal disease in patients with pT1–2 (pN1: OR 2·72, 1·35 to 5·48; pN2–3: OR 5·00, 2·35 to 10·66) or pT3–4 (pN1: OR 3·03, 1·51 to 6·07; pN2–3: OR 5·75, 3·15 to 10·49) disease predisposed to systemic recurrence. Poor or no response to chemotherapy was also an independent risk factor for isolated systemic recurrence (OR 1·85, 1·05 to 3·26). A positive resection margin (R1 resection) was not associated with a significantly increased risk of isolated locoregional recurrence (OR 1·37, 0·81 to 2·33).

**Conclusion:**

These findings confirm that oesophageal adenocarcinoma is frequently a systemic disease. Understanding the key predictors of local and systemic recurrence may facilitate the tailoring of oncological therapies to the individual patient.

## Introduction

Oesophageal cancer is the sixth most common cancer worldwide and is responsible for 400 000 deaths a year^1^. Once the disease has progressed beyond the mucosa, oesophagectomy is generally an important element in any treatment protocol designed to achieve cure. Unfortunately, a high proportion of patients have evidence of micrometastasis at the time of surgery, and half of all resected patients develop recurrent disease within 2 years of surgery^2^, ^3^, ^4^, ^5^, ^6^. Systemic recurrence remains the most common cause of death following oesophageal resection and, as a result, most patients are offered oncological therapies in combination with surgery, in the hope of reducing this risk^7^. Neoadjuvant chemotherapy (NAC) and neoadjuvant chemoradiotherapy (NACRT) have both been shown to improve survival compared with surgery alone^7^, ^8^, ^9^. Although both may have a local downstaging effect on the primary tumour, this is widely acknowledged to be more pronounced following NACRT^8^. Debate still exists regarding whether this local benefit of NACRT is at the cost of reduced systemic efficacy compared with NAC^9^.

Understanding patterns of recurrence of oesophageal cancer after surgery may be useful in stratifying patients to oncological treatment alternatives and informing future trials. This study was designed to identify clinicopathological factors associated with locoregional and systemic recurrence in oesophageal adenocarcinoma.

## Methods

This was a cohort study based on a prospectively developed database of consecutive resections performed at Guy's and St Thomas' Oesophago‐Gastric Centre, London, UK. The study involved all patients who underwent oesophagectomy between 2000 and 2014 for adenocarcinoma or squamous cell carcinoma (SCC). Patients with Siewert type III junctional tumours having NACRT and those undergoing oesophagogastrectomy for benign or rare malignant pathologies (melanoma, sarcoma and neuroendocrine tumours) were excluded. The main outcome measure was the presence of tumour recurrence. Other outcome measures were time to recurrence and survival. Follow‐up ended in February 2016.

### Clinical management

Patients underwent a standard protocol of investigations including oesophagogastroduodenoscopy, CT, endoscopic ultrasonography and, from 2007, fluorodeoxyglucose‐PET. The practice of NAC evolved during the study period and followed standard indications and regimens, as supported by RCT evidence^9^. Surgical resection included transthoracic (TTO) or transhiatal (THO) oesophagectomy, determined by tumour characteristics and individual surgeon preference. Histological staging was standardized to meet the seventh edition of TNM criteria. Pathological specimens were processed and reported using the Royal College of Pathologists' guidelines. A positive circumferential resection margin (CRM) was defined as tumour within 1 mm of the cut margin. Adjuvant therapy was determined by the multidisciplinary team (MDT), based on the positivity of resection margins, pathological nodal status and the postoperative performance status of the patient.

### Tumour recurrence criteria

Tumour recurrences were classified as either locoregional or systemic, and were diagnosed radiologically or histologically with MDT consensus. Locoregional recurrence was further subcategorized into regional lymph node, mediastinal mass, abdominal mass or anastomotic recurrence. Regional lymph nodes included mediastinal, left gastric and coeliac nodes for patients with gastro‐oesophageal junction tumours, defined on the basis that they were within the lymphatic distribution of the primary tumour and fell inside a therapeutic radiotherapy field. Mediastinal and abdominal recurrences represented mass recurrences in the original tumour bed, acknowledging some inevitable overlap with the local lymph node group. Anastomotic recurrences were defined as intraluminal disease on endoscopy, confirmed histologically.

Systemic recurrence was divided into haematogenous, distant lymph node and peritoneal recurrences. Haematogenous recurrences included lung, liver, bone, adrenal and brain. Distant nodal metastases included supraclavicular, para‐aortic, portal and mesenteric nodes considered to be outside a conventional radiotherapy or surgical field. Peritoneal disease was examined independently as it was considered to represent a separate (transcoelomic) mode of dissemination.

In all, six outcome groups were examined: no recurrence, any recurrence, locoregional recurrence, systemic recurrence, isolated locoregional recurrence and isolated systemic recurrence. The locoregional group included patients who experienced isolated locoregional recurrence plus those who had locoregional recurrence as part of a mixed pattern. This distinction was made to analyse the risk of developing any local recurrence. The same applied to the systemic recurrence group. Whether local recurrence occurred in isolation or as part of a systemic recurrence, it still represented a failure of local control. Isolated recurrence was defined as a situation with no evidence of metachronous recurrence within 6 weeks. If a second recurrence was confirmed within 6 weeks of the first, it was deemed synchronous^10^.

### Statistical analysis

To evaluate which patient and tumour characteristics were predictive of each recurrence type, crude logistic regression analysis was performed first. Multivariable logistic regression with backward stepwise elimination (α = 0·20) was then used to identify individual predictors of recurrence. Only the adenocarcinoma subgroup had sufficient numbers to be included in the statistical models. Patient and tumour characteristics examined were: sex (male or female), age (continuous), preoperative stenting (yes or no), NAC (yes or no), surgery type (TTO *versus* THO), resection outcome (R0 or R1), lymphovascular invasion (yes or no), pathological stage (pT0 N0, pT1–2 N0, pT1–2 N1, pT1–2 N2–3, pT3–4 N0, pT3–4 N1, pT3–4 N2–3), pathological grade (poorly differentiated, moderately differentiated, well differentiated or complete pathological response), Mandard tumour regression score (1, 2–3, 4–5, or not applicable) and adjuvant treatment (none, chemotherapy or chemoradiotherapy). Time to recurrence was considered to be less relevant, particularly as most recurrences after oesophagectomy occur within 2 years^4^, ^5^, ^6^. It was therefore decided to treat the outcome ‘recurrence’ as a categorical variable using logistic regression. To verify this assumption, an additional Cox regression analysis using time to recurrence as the primary outcome was performed.

## Results

Of 761 consecutive patients who underwent oesophagectomy between 2000 and 2014, 698 with adenocarcinoma (578, 82·8 per cent) or SCC (120, 17·2 per cent) were identified. Their clinicopathological features are shown in *Table* 1.

**Table 1:** Demographics and clinical characteristics of participating patients with oesophageal cancer according to histological type

	Adenocarcinoma (*n* = 578)	Squamous cell carcinoma (*n* = 120)
Mean age (years)	62·5	62·4
Sex ratio (M : F)	494 : 84	56 : 64
Neoadjuvant treatment		
None	153 (26·5)	57 (47·5)
Chemotherapy	424 (73·4)	63 (52·5)
Missing	1 (0·2)	
Surgery		
Transthoracic	281 (48·6)	72 (60·0)
Transhiatal	297 (51·4)	48 (40·0)
Pathological stage		
T0 N0	22 (3·8)	11 (9·2)
T1–2 N0	123 (21·3)	12 (10·0)
T1–2 N1	66 (11·4)	38 (31·7)
T1–2 N2–3	49 (8·5)	8 (6·7)
T3–4 N0	74 (12·8)	2 (1·7)
T3–4 N1	70 (12·1)	25 (20·8)
T3–4 N2–3	161 (27·9)	16 (13·3)
Missing	13 (2·2)	8 (6·7)
Pathological grade		
Poorly differentiated	220 (38·1)	31 (25·8)
Moderately differentiated	311 (53·8)	66 (55·0)
Well differentiated	13 (2·2)	8 (6·7)
Complete pathological response	22 (3·8)	11 (9·2)
Missing	12 (2·1)	4 (3·3)
Response to chemotherapy		
Mandard 1 (complete pathological response)	22 (3·8)	11 (9·2)
Mandard 2 (good response)	18 (3·1)	3 (2·5)
Mandard 3 (moderate response)	126 (21·8)	15 (12·5)
Mandard 4 (poor response)	194 (33·6)	27 (22·5)
Mandard 5 (no response)	39 (6·7)	4 (3·3)
n.a.	153 (26·5)	57 (47·5)
Missing	26 (4·5)	3 (2·5)
Resection margin		
R0	303 (52·4)	77 (64·2)
R1	275 (47·6)	43 (35·8)
CRM within 1 mm of margin (UK)	272 (47·1)	43 (35·8)
CRM tumour at margin (USA)	104 (18·0)	13 (10·8)
Longitudinal margin positive	42 (7·3)	1 (0·8)
Lymphovascular invasion		
Yes	309 (53·5)	42 (35·0)
No	266 (46·0)	78 (65·0)
Missing	3 (0·5)	0 (0)
Two‐field positive lymph nodes		
Yes	140 (24·2)	13 (10·8)
No	438 (75·8)	107 (89·2)
Adjuvant treatment		
None	254 (43·9)	69 (57·5)
Chemotherapy	202 (34·9)	29 (24·2)
Chemoradiotherapy	99 (17·1)	18 (15·0)
Missing	23 (4·0)	4 (3·3)

Values in parentheses are percentages. n.a., not applicable; CRM, circumferential resection margin.

The mean age of patients was 62·5 years. The male to female ratio was higher for adenocarcinoma (494 : 84) than for SCC (64 : 56). In the adenocarcinoma group, 73·4 per cent of patients underwent NAC, a proportion that increased during the study interval as thresholds for NAC lowered. Of the 153 who proceeded straight to surgery, 98 (64·1 per cent) had T1–2 disease and 107 (69·9 per cent) had no nodal disease on preoperative staging.

Twenty‐five patients, all with T3 disease, had stenting before surgery; all but one received NAC. Fourteen of these 25 patients had an R1 resection and eight were downstaged by NAC (5 to pT2; 3 to pT1).

### Oncological outcomes

Overall recurrence‐free survival of the cohort was 75·4, 53·1 and 45·2 per cent at 1, 3 and 5 years respectively. R1 resection rates were higher in the adenocarcinoma cohort (47·6 per cent) than in patients with SCC (35·8 per cent). CRM rates overall were 45·1 per cent. Longitudinal margin involvement in isolation was rare (2·2 per cent). Median follow‐up was 1·62 (range 0·01 to 13·84) years.

### Recurrence patterns

Of the 698 patients, 326 (46·7 per cent) developed recurrence. Patterns of recurrence according to histological subtype are shown in *Table* 2. There was a higher rate of overall recurrence for adenocarcinoma compared with SCC (48·6 *versus* 37·5 per cent respectively), despite a higher rate of NAC in the adenocarcinoma group (73·4 *versus* 52·5 per cent) (*Table* 1). This was due mainly to a higher rate of systemic recurrence in the adenocarcinoma group (37·7 *versus* 22·8 per cent; *P* = 0·013). In all, 27·0 per cent of patients with adenocarcinoma developed local recurrence, compared with 22·5 per cent of patients with SCC. However, many of these (16·3 per cent for adenocarcinoma and 10·8 per cent for SCC) were associated with synchronous systemic metastases. Rates of isolated locoregional recurrence were 10·7 and 11·7 per cent respectively.

**Table 2:** Distribution of local and systemic recurrence following surgery for oesophageal cancer according to histology and neoadjuvant chemotherapy groups

	Adenocarcinoma*			Squamous cell carcinoma		
	All (*n* = 578)	NAC (*n* = 424)	No NAC (*n* = 153)	All (*n* = 120)	NAC (*n* = 63)	No NAC (*n* = 57)
Recurrence						
No	297 (51·4)	202 (47·6)	93 (60·8)	75 (62·5)	40 (63)	35 (61)
Yes	281 (48·6)	222 (52·4)	60 (39·2)	45 (37·5)	23 (37)	22 (39)
Local recurrence	156 (27·0)	121 (28·5)	35 (22·9)	27 (22·5)	16 (25)	11 (19)
Isolated	62 (10·7)	47 (11·1)	15 (9·8)	14 (11·7)	9 (14)	5 (9)
Anastomotic	34 (5·9)	23 (5·4)	11 (7·2)	14 (11·7)	8 (13)	6 (11)
Lymph node	85 (14·7)	67 (15·8)	18 (11·8)	8 (6·7)	6 (10)	2 (4)
Mediastinal nodal	61 (10·6)	48 (11·3)	13 (8·5)	6 (5·0)	4 (6)	2 (4)
Left gastric nodal	7 (1·2)	6 (1·4)	1 (0·7)	0 (0)	0 (0)	0 (0)
Coeliac nodal	31 (5·4)	26 (6·1)	5 (3·3)	2 (1·7)	2 (3)	0 (0)
Abdominal	3 (0·5)	3 (0·7)	0 (0)	2 (1·7)	2 (3)	0 (0)
Mediastinal	35 (6·1)	26 (6·1)	9 (5·9)	7 (5·8)	4 (6)	3 (5)
Systemic recurrence	218 (37·7)	174 (41·0)	44 (28·8)	31 (25·8)	17 (27)	17 (30)
Isolated	124 (21·5)	47 (11·1)	23 (15·0)	18 (15·0)	7 (11)	11 (19)
Haematogenous	118 (20·4)	96 (22·6)	22 (14·4)	15 (12·5)	8 (13)	7 (12)
Lung	68 (11·8)	52 (12·3)	16 (10·5)	13 (10·8)	6 (10)	7 (12)
Liver	67 (11·6)	52 (12·3)	15 (9·8)	14 (11·7)	7 (11)	7 (12)
Bone	38 (6·6)	31 (7·3)	7 (4·6)	3 (2·5)	2 (3)	1 (2)
Brain	14 (2·4)	13 (3·1)	1 (0·7)	0 (0)	0 (0)	0 (0)
Nodal	39 (6·7)	31 (7·3)	8 (5·2)	8 (6·7)	4 (6)	4 (7)
Supraclavicular	10 (1·7)	6 (1·4)	4 (2·6)	4 (3·3)	1 (2)	3 (5)
Para‐aortic	13 (2·2)	12 (2·8)	1 (0·7)	3 (2·5)	3 (5)	0 (0)
Mesenteric	5 (0·9)	5 (1·2)	0 (0)	0 (0)	0 (0)	0 (0)
Porta hepatis	5 (0·9)	3 (0·7)	2 (1·3)	1 (0·8)	0 (0)	1 (2)
Other	3 (0·5)	2 (0·5)	1 (0·7)	0 (0)	0 (0)	0 (0)
Peritoneal	54 (9·3)	43 (10·1)	11 (7·2)	5 (4·2)	5 (8)	0 (0)

Values in parentheses are percentages.

* As shown in *Table* 1, information on neoadjuvant chemotherapy (NAC) was missing for one patient.

There was a higher rate of haematogenous metastasis in patients with adenocarcinoma than in those with SCC (20·4 *versus* 12·5 per cent respectively). Although 30·5 per cent (64 of 210) of patients with pN2–3 adenocarcinoma developed haematogenous recurrence, this also occurred in 11·9 per cent (26 of 219) of patients with pN0 and 23·5 per cent (32 of 136) of those with pN1 disease. Of the 318 patients with an R1 resection, 194 (61·0 per cent) suffered recurrent disease, which was isolated locoregional recurrence in only 42 patients (13·2 per cent). Most of the patients with recurrence after R1 resection (150 of 194, 77·3 per cent) developed systemic recurrence. There appeared to be no correlation between Siewert type I and II junctional tumours regarding rates of peritoneal recurrence: 26 of 261 (10·0 per cent) and 30 of 290 (10·3 per cent) respectively. Most patients (21 of 33, 64 per cent) who had coeliac lymph node recurrence were found to have synchronous systemic disease. Thirty‐three patients had a complete pathological response to NAC. There were no recorded recurrences among these patients. Systemic recurrence rates in patients with adenocarcinoma who had undergone NAC were 18·9 per cent (28 of 148) for pN0, 44·5 per cent (49 of 110) for pN1, 57 per cent (51 of 89) for pN2 and 60 per cent (46 of 77) for pN3 disease.

### Factors associated with systemic and locoregional recurrence in adenocarcinoma

Crude and multivariable analysis of recurrence patterns was performed only in patients with adenocarcinoma (*Tables* 3 and 4). In crude analysis, there was an association between nodal status and the risk of all types of recurrence, particularly pT3–4 N2–3 disease (any recurrence: OR 8·41, 95 per cent c.i. 4·91 to 14·40; locoregional recurrence: OR 3·85, 2·13 to 6·95; systemic recurrence: OR 6·09, 3·53 to 10·52; isolated locoregional recurrence: OR 2·76, 1·14 to 6·67; isolated systemic recurrence: OR 4·27, 2·20 to 8·27). Lymphovascular invasion was associated with all forms of recurrence, and stenting was associated with locoregional recurrence (OR 3·06, 1·25 to 7·53) and isolated locoregional recurrence (OR 3·83, 1·41 to 10·38). Mandard score 4–5 (poor or no response) was associated with an increased risk of systemic (OR 2·07, 1·34 to 3·20) and isolated systemic (OR 2·33, 1·37 to 3·98) recurrence.

**Table 3:** Crude odds ratios for risk of recurrence in patients with oesophageal adenocarcinoma

	Odds ratio				
	Any recurrence	Locoregional recurrence	Systemic recurrence	Isolated locoregional recurrence	Isolated systemic recurrence
Age at operation	0·98 (0·96, 1·00)	0·99 (0·97, 1·01)	0·98 (0·96, 1·00)	1·00 (0·97, 1·03)	0·99 (0·97, 1·01)
Sex					
M	1·39 (0·87, 2·22)	1·42 (0·81, 2·48)	1·04 (0·64, 1·68)	2·66 (0·94, 7·53)	1·09 (0·61, 1·93)
F	1·00 (reference)	1·00 (reference)	1·00 (reference)	1·00 (reference)	1·00 (reference)
Initial stage					
T1–2 N0	1·00 (reference)	1·00 (reference)	1·00 (reference)	1·00 (reference)	1·00 (reference)
T1–2 N+	3·05 (1·48, 6·26)	2·93 (1·25, 6·87)	3·02 (1·39, 6·55)	1·29 (0·37, 4·45)	1·83 (0·72, 4·66)
T3–4 N0	3·79 (2·22, 6·49)	2·99 (1·52, 5·85)	3·66 (2·02, 6·65)	1·71 (0·70, 4·18)	2·46 (1·22, 4·97)
T3–4 N+	3·30 (1·69, 6·45)	2·62 (1·16, 5·88)	2·78 (1·34, 5·76)	2·26 (0·79, 6·45)	2·20 (0·94, 5·16)
Tumour location					
Lower oesophagus	1·00 (reference)	1·00 (reference)	1·00 (reference)	1·00 (reference)	1·00 (reference)
Siewert type I	2·36 (1·14, 4·89)	3·00 (1·13, 7·96)	2·45 (1·08, 5·56)	1·19 (0·39, 3·57)	1·17 (0·49, 2·81)
Siewert type II	2·00 (0·97, 4·13)	2·25 (0·85, 5·99)	2·35 (1·04, 5·31)	0·90 (0·30, 2·72)	1·27 (0·53, 3·02)
Preoperative stenting					
Yes	6·88 (1·99, 23·76)	3·06 (1·25, 7·53)	2·28 (0·93, 5·61)	3·83 (1·41, 10·38)	2·08 (0·81, 5·35)
No	1·00 (reference)	1·00 (reference)	1·00 (reference)	1·00 (reference)	1·00 (reference)
Neoadjuvant chemotherapy					
Yes	1·73 (1·19, 2·53)	1·33 (0·87, 2·06)	1·71 (1·14, 2·55)	1·14 (0·62, 2·10)	1·75 (1·07, 2·88)
No	1·00 (reference)	1·00 (reference)	1·00 (reference)	1·00 (reference)	1·00 (reference)
Type of surgery					
TTO	1·49 (1·07, 2·07)	1·38 (0·95, 2·00)	1·23 (0·88, 1·72)	1·65 (0·97, 2·82)	1·21 (0·82, 1·81)
THO	1·00 (reference)	1·00 (reference)	1·00 (reference)	1·00 (reference)	1·00 (reference)
Type of resection					
R0	1·00 (reference)	1·00 (reference)	1·00 (reference)	1·00 (reference)	1·00 (reference)
R1	2·68 (1·91, 3·75)	1·86 (1·28, 2·70)	2·53 (1·79, 3·57)	1·37 (0·81, 2·33)	2·01 (1·34, 3·02)
Lymphovascular invasion					
Yes	3·26 (2·32, 4·60)	2·35 (1·59, 3·46)	2·66 (1·87, 3·79)	2·10 (1·19, 3·70)	2·11 (1·39, 3·21)
No	1·00 (reference)	1·00 (reference)	1·00 (reference)	1·00 (reference)	1·00 (reference)
Pathological staging					
pT0 N0	0·10 (0·01, 0·78)	–	0·14 (0·02, 1·10)	–	0·29 (0·04, 2·32)
pT1–2 N0	1·00 (reference)	1·00 (reference)	1·00 (reference)	1·00 (reference)	1·00 (reference)
pT1–2 N1	3·35 (1·78, 6·30)	2·36 (1·14, 4·90)	2·68 (1·38, 5·22)	2·29 (0·79, 6·61)	2·71 (1·21, 6·05)
pT1–2 N2–3	4·30 (2·14, 8·67)	1·89 (0·83, 4·30)	4·30 (2·10, 8·79)	1·47 (0·41, 5·28)	4·50 (1·98, 10·23)
pT3–4 N0	1·26 (0·66, 2·39)	1·24 (0·57, 2·71)	0·96 (0·46, 2·00)	2·01 (0·70, 5·79)	1·17 (0·48, 2·89)
pT3–4 N1	5·02 (2·67, 9·46)	4·38 (2·20, 8·71)	3·47 (1·82, 6·64)	3·43 (1·28, 9·17)	2·12 (0·93, 4·81)
pT3–4 N2–3	8·41 (4·91, 14·40)	3·85 (2·13, 6·95)	6·09 (3·53, 10·52)	2·76 (1·14, 6·67)	4·27 (2·20, 8·27)
Pathological grade					
Poorly differentiated	1·00 (reference)	1·00 (reference)	1·00 (reference)	1·00 (reference)	1·00 (reference)
Moderately differentiated	0·82 (0·58, 1·16)	0·81 (0·55, 1·18)	0·90 (0·63, 1·28)	0·76 (0·44, 1·29)	0·99 (0·65, 1·49)
Well differentiated	0·71 (0·23, 2·19)	0·66 (0·18, 2·46)	0·89 (0·28, 2·80)	0·55 (0·07, 4·38)	1·02 (0·27, 3·85)
Complete pathological response	–	–	–	–	–
Two‐field positive lymph nodes					
Yes	1·70 (1·16, 2·50)	1·11 (0·73, 1·70)	1·61 (1·10, 2·37)	1·21 (0·67, 2·19)	1·86 (1·21, 2·87)
No	1·00 (reference)	1·00 (reference)	1·00 (reference)	1·00 (reference)	1·00 (reference)
Mandard score					
1	–	–	–	–	–
2–3	1·00 (reference)	1·00 (reference)	1·00 (reference)	1·00 (reference)	1·00 (reference)
4–5	2·16 (1·42, 3·30)	1·25 (0·79, 1·98)	2·07 (1·34, 3·20)	1·14 (0·59, 2·18)	2·33 (1·37, 3·98)
n.a.	1·05 (0·67, 1·63)	0·86 (0·52, 1·43)	1·13 (0·71, 1·81)	0·85 (0·41, 1·75)	1·27 (0·74, 2·30)
Adjuvant treatment					
None	1·00 (reference)	1·00 (reference)	1·00 (reference)	1·00 (reference)	1·00 (reference)
Chemotherapy	1·79 (1·24, 2·61)	2·19 (1·45, 3·33)	1·49 (1·02, 2·19)	1·63 (0·90, 2·95)	0·89 (0·56, 1·43)

Values in parentheses are 95 per cent confidence intervals. TTO, transthoracic oesophagectomy; THO, transhiatal oesophagectomy; n.a., not applicable.

**Table 4:** Multivariable odds ratios for risk of recurrence in the patients with oesophageal adenocarcinoma

	Odds ratio				
	Any recurrence	Locoregional recurrence	Systemic recurrence	Isolated locoregional recurrence	Isolated systemic recurrence
Sex					
M	1·49 (0·87, 2·57)			2·77 (0·97, 7·96)	
F	1·00 (reference)			1·00 (reference)	
Preoperative tumour grade					
Poorly differentiated			1·00 (reference)		
Moderately differentiated			0·90 (0·88, 2·09)		
Well differentiated			1·99 (0·23, 3·47)		
Preoperative stenting					
Yes	5·48 (1·43, 21·08)	2·88 (1·10, 7·51)	1·49 (0·56, 4·02)	3·70 (1·34, 10·23)	
No	1·00 (reference)	1·00 (reference)	1·00 (reference)	1·00 (reference)	
Neoadjuvant chemotherapy					
Yes	1·58 (1·00, 2·50)		1·90 (1·17, 3·08)		3·08 (1·24, 7·67)
No	1·00 (reference)		1·00 (reference)		1·00 (reference)
Lymphovascular invasion					
Yes	1·54 (1·02, 2·35)			2·09 (1·18, 3·71)	
No	1·00 (reference)			1·00 (reference)	
Pathological staging					
pT0 N0	0·13 (0·02, 1·03)	–	0·14 (0·02, 1·13)		1·31 (0·15, 11·64)
PT1–2 N0	1·00 (reference)	1·00 (reference)	1·00 (reference)		1·00 (reference)
pT1–2 N1	2·98 (1·51, 5·86)	2·08 (0·98, 4·42)	2·72 (1·35, 5·48)		2·54 (1·22, 5·74)
pT1–2 N2–3	4·10 (1·93, 8·71)	1·72 (0·74, 4·03)	5·00 (2·35, 10·66)		4·17 (1·81, 9·58)
pT3–4 N0	1·09 (0·54, 2·18)	1·18 (0·53, 2·63)	0·79 (0·37, 1·72)		0·98 (0·39, 2·47)
pT3–4 N1	3·80 (1·89, 7·65)	4·01 (1·95, 8·26)	3·03 (1·51, 6·07)		1·75 (0·76, 4·05)
pT3–4 N2–3	6·26 (3·34, 11·73)	3·78 (2·02, 7·06)	5·75 (3·15, 10·49)		3·12 (1·56, 6·24)
Mandard score					
1					–
2–3					1·00 (reference)
4–5					1·85 (1·05, 3·26)
n.a.					2·97 (1·18, 7·43)
Adjuvant treatment					
None	1·00 (reference)	1·00 (reference)	1·00 (reference)		
Chemotherapy	1·19 (0·77, 1·86)	1·75 (1·11, 2·75)	1·04 (0·67, 1·62)		
Chemoradiotherapy	1·27 (0·71, 2·27)	0·98 (0·54, 1·77)	1·32 (0·75, 2·32)		

The model was based on backward elimination with α = 0·20 and exclusion of initial stage, two‐field positive nodes and tumour location. Values in parentheses are 95 per cent confidence intervals. n.a., not applicable.

In multivariable analysis, lymphovascular invasion (OR 2·09, 95 per cent c.i. 1·18 to 3·71) and preoperative stenting (OR 3·70, 1·34 to 10·23) were independent risk factors for isolated locoregional recurrence. Advanced pathological stage with pT3–4 N1 (OR 4·01, 1·95 to 8·26) and pT3–4 N2–3 (OR 3·78, 2·02 to 7·06) disease was a risk factor for locoregional recurrence.

Poor or no response to chemotherapy (Mandard score 4–5: OR 1·85, 95 per cent c.i. 1·05 to 3·26) was an independent risk factor for isolated systemic recurrence. Pathological nodal status was also an independent risk factor for systemic recurrence, in patients with both pT1–2 (pN1: OR 2·72, 1·35 to 5·48; pN2–3: OR 5·00, 2·35 to 10·66) and T3–4 (pN1: OR 3·03, 1·51 to 6·07; pN2–3: OR 5·75, 3·15 to 10·49) disease.

In univariable analysis, R1 resection was associated with higher rates of overall (OR 2·68, 95 per cent c.i. 1·91 to 3·75), locoregional (OR 1·86, 1·28 to 2·70) and systemic (OR 2·53, 1·79 to 3·57) recurrence, but not isolated locoregional recurrence (OR 1·37, 0·81 to 2·33). An R1 resection did not increase risk of any recurrence type on multivariable analysis.

## Discussion

This study has indicated that lymphovascular invasion and preoperative stenting are independently associated with isolated locoregional recurrence after oesophagectomy for adenocarcinoma, whereas advanced nodal disease and a poor response to chemotherapy predict systemic recurrence. Patients with adenocarcinoma had a higher rate of systemic recurrence than those with SCC. A positive resection margin did not lead to significantly higher rates of local recurrence, and the majority of these patients died from systemic disease. In contrast to other series^11^ ^12^, this study did not show a higher rate of local recurrence in the SCC group.

Some methodological issues deserve attention. Although many previous studies have examined recurrence patterns following oesophagectomy alone, few have assessed this question in an era when NAC has been used routinely. Given the current debate on the optimal perioperative treatment of oesophageal adenocarcinoma, the pattern of disease recurrence is an important issue. This was a relatively large study in terms of patient numbers, from a single centre with mature follow‐up data. Analysis of adenocarcinoma and SCC separately reduced the heterogeneity of the groups. Although patient numbers allowed adjustment for several confounding factors, the retrospective nature of the study and the evolution of perioperative treatment strategies over the study period were sources of potential bias. Some confounders such as advanced T status, stenting and positive resection margins had strong interactions.

There is little consensus as to how locoregional and systemic recurrences should be categorized. Intuitively, locoregional recurrences would best be defined as those occurring within an agreed target area of a given locoregional therapy, either a field of surgical resection with lymphadenectomy or radiotherapy. Previous studies 12, 13 have used both criteria but with areas of notable contention such as the inclusion of supraclavicular lymph nodes as locoregional recurrence for SCC^12^. Equally contentious in the context of adenocarcinoma is the status of coeliac lymph nodes, which were traditionally considered M1a disease in the sixth TNM classification. These nodes are variably included by surgeons and oncologists in lymphadenectomy and radiotherapy fields. Interestingly, the present data showed high rates of coexisting systemic recurrence in patients with coeliac lymph node recurrence, as found elsewhere^12^. This implies that locoregional control is not the predominant issue in patients with involved coeliac nodes. It is also important to recognize that mode of spread may vary between patients, and although haematogenous, distant lymph node and peritoneal recurrences were all classified as distant metastases, the risk factors for each of these modes of distribution may be different. An understanding of this may guide future oncological therapeutic strategies.

Pathological nodal status is a known marker of recurrence and prognosis. One multicentre study^13^ analysed 1053 patients, and demonstrated that the risk of systemic recurrence increased with pathological nodal status in patients progressing straight to surgery without NAC (pN0, 16 per cent; pN1, 44 per cent; pN2, 69 per cent; pN3, 93 per cent; *P* < 0·001). The present study found similar systemic recurrence rates in patients with pN0, pN1 and pN2 adenocarcinoma who had undergone NAC; however, the systemic recurrence rate in patients with pN3 disease was 60 per cent. Although NAC might reduce the systemic recurrence rate in patients with more advanced nodal disease, there is little evidence to support a major effect in patients who seem to have a more favourable disease stage.

The finding that patients with a poor response to NAC have double the risk of systemic recurrence compared with those with a good or moderate response is inherently logical. In keeping with this, a recent study^14^ found no survival benefit for adjuvant platinum‐based chemotherapy in patients who were non‐responders in the neoadjuvant setting. Prospective trials are required to determine any benefit of changing or tailoring chemotherapy regimens in non‐responding patients.

The present study confirmed that the incidence of isolated locoregional recurrence within a surgical or radiotherapy field is rare. Even in patients who might be considered at high risk for isolated locoregional recurrence, such as those with a positive resection margin, recurrence occurs in a predominantly systemic fashion. A previous study^12^ documented the same finding after NACRT, with isolated locoregional recurrence being found in only 9 and 3 per cent respectively of patients receiving surgery alone and those who had NACRT. Most patients developed systemic recurrence following NACRT. In that same study^12^, lymph node‐positive patients and those who did not receive neoadjuvant treatment had an increased risk of local recurrence, but the benefit of treatment was greatest in the SCC group. Given that the outcomes of most patients with adenocarcinoma will be dictated by the presence of systemic disease, the role for radiotherapy as standard practice in all patients should still be questioned, particularly as this is often accompanied by a reduction in systemic chemotherapy dose. This is indirectly supported by follow‐up data of complete responders to NACRT, who continue to suffer significant rates of systemic relapse despite having no residual tumour at the time of resection^12^. In contrast, none of the patients who showed a complete pathological response following NAC in the present series had a recurrence.

Preoperative stenting remains a contentious issue. In the present series, one‐third of stented patients were downstaged following NAC. Despite this, stenting remained a significant independent risk factor for locoregional recurrence. Whether this was simply a reflection of a locally advanced tumour or whether the stenting itself predisposed to recurrence by expanding the tumour towards its lateral margins is unclear. Although stenting poses challenges for radiotherapy field planning, the higher rates of local recurrence in stented patients might suggest that this group be given particular consideration for radiotherapy before or after surgery.

Lymphovascular invasion is a known prognostic factor in both SCC and adenocarcinoma^15^ ^16^, and has been associated with a higher risk of recurrence^17^. In the present study, lymphovascular invasion was an independent risk factor for overall recurrence and isolated locoregional recurrence. The rate of lymphovascular invasion in the adenocarcinoma group was 53·5 per cent, compared with 35·0 per cent in the SCC group, in line with other studies^15^ ^16^.

This study indicates that adenocarcinoma and SCC have differing pathophysiology. Trials that involve both histopathologies should take this into account. Understanding how clinicopathological factors influence recurrence patterns in both main histological types of oesophageal cancer may be useful in creating tailored neoadjuvant and adjuvant treatment pathways. The presence of lymphovascular invasion, stenting and pT3–4 with node‐positive disease predisposes to local recurrence in adenocarcinoma. Nodal status and poor response to chemotherapy predict systemic recurrence. As staging modalities become more sensitive, trials will be needed to determine whether treatment strategies based on risk of recurrence will yield improvements in long‐term survival.
